# PGMD/curcumin nanoparticles for the treatment of breast cancer

**DOI:** 10.1038/s41598-021-81701-x

**Published:** 2021-02-15

**Authors:** Mankamna Kumari, Nikita Sharma, Romila Manchanda, Nidhi Gupta, Asad Syed, Ali H. Bahkali, Surendra Nimesh

**Affiliations:** 1 Department of Biotechnology, School of Life Sciences, Central University of Rajasthan, Bandarsindri, N.H. 8, Teh., Kishangarh, Dist., Ajmer, Rajasthan 305817 India; 2 School of Basic and Applied Sciences, K.R. Mangalam University, Sohna Road, Gurugram, 122103 India; 3 Department of Biotechnology, IIS (Deemed To Be University), Gurukul Marg, SFS, Mansarovar, Jaipur, Rajasthan 302020 India; 4 Department of Botany and Microbiology, College of Science, King Saud University, P.O. 2455, Riyadh, 11451 Saudi Arabia

**Keywords:** Biotechnology, Nanoscience and technology

## Abstract

The present study aims at developing PGMD (poly-glycerol-malic acid-dodecanedioic acid)/curcumin nanoparticles based formulation for anticancer activity against breast cancer cells. The nanoparticles were prepared using both the variants of PGMD polymer (PGMD 7:3 and PGMD 6:4) with curcumin (i.e. CUR NP 7:3 and CUR NP 6:4). The size of CUR NP 7:3 and CUR NP 6:4 were found to be ~ 110 and 218 nm with a polydispersity index of 0.174 and 0.36, respectively. Further, the zeta potential of the particles was − 18.9 and − 17.5 mV for CUR NP 7:3 and CUR NP 6:4, respectively. The entrapment efficiency of both the nanoparticles was in the range of 75–81%. In vitro anticancer activity and the scratch assay were conducted on breast cancer cell lines, MCF-7 and MDA-MB-231. The IC_50_ of the nanoformulations was observed to be 40.2 and 33.6 μM at 48 h for CUR NP 7:3 and CUR NP 6:4, respectively, in MCF-7 cell line; for MDA-MB-231 it was 43.4 and 30.5 μM. Acridine orange/EtBr and DAPI staining assays showed apoptotic features and nuclear anomalies in the treated cells. This was further confirmed by western blot analysis that showed overexpression of caspase 9 indicating curcumin role in apoptosis.

## Introduction

With the changing environmental factors and lifestyle such as pollution, tobacco smoking, diet patterns, there has been a tremendous increase in the incidence of cancer. This makes it the second most leading cause of death after cardiovascular disease^1^. Amongst different types of cancers, breast cancer is known to be the leading cause of morbidity and mortality in women with around 450,000 deaths around the world^2^. The breast cancer is divided on the basis of presence or overexpression of estrogen, progesterone and HER-2 receptors. Further, the breast cancer cells could also be triple-negative that lacks any of the receptors. While, the receptor positive cells are treated by receptor-specific therapy, chemotherapy remains to be the only regimen for triple-negative breast cancer cells^3^. Thus, TNBC presents therapeutic challenge, as it responds minimally to the hormone-based therapy and higher chance of cancer cell dissemination and development of brain metastasis^4, 5^. Hence, despite of current modalities for treatment of cancer there are certain side effects that can be life-threatening^6^. Further, the available anticancer drugs have poor pharmacokinetic properties; poor solubility, stability, metabolism, biodistribution and toxicity. This instigates the necessity to explore effective formulations that can overcome the above cited challenges and provide targeted delivery to avoid damages to normal tissues or cells^7–9^.

Nanomedicine has received extensive interest in the journey of diagnostics and therapeutics owing to their small size, beneficial and tunable properties. Nanoparticles are submicron (100–1000 nm) sized particles that are generally synthesized from materials such as polymers, lipids, virus and inorganic materials^10–17^. Several polymers, such as poly (lactic-*co*-glycolic acid) (PLGA), poly (lactic acid) (PLA) etc. have been vividly used for cancer treatment, where few formulations also gained approval by regulatory agencies. Some of the nanotherapeutics used for the treatment of metastatic breast cancer are: Abraxane (albumin-based, paclitaxel nanoparticle formulation)^18, 19^. Previous studies have shown that therapeutic agents gets cleared and degraded causing undesirable biological behaviour and toxicities^20, 21^. Thus nanoparticles possess certain limitations such as poor bioavailability, instability, toxicity and inadequate tissue distribution. Also, on a survey of 10 years of studies on nanoparticles based drug delivery systems, it showed that only 0.7% of the administered drug formulations were capable enough to reach the solid tumors. Thus, it becomes imperative to develop efficient nanoformulations with lower side effects^22^.

For the past few decades, there is a tremendous increase in the use of natural products due to undesirable properties and side effects of synthetic drugs. It is believed that natural compounds would be well tolerated in the treatment of different ailments. Some of the compounds such as epigallocatechin gallate (EGCG), Sulphoraphane and resveratrol have shown promising activity on Breast cancer cells. EGCG, a well known phenolic catechin in green tea has shown to promote apoptosis in Breast cancer cells by inducing overexpression of pro-apoptotic genes, such as caspase 3, 8 and 9. Similarly, sulphoraphane showed inhibition of hTERT (human telomerase reverse transcriptase) in a dose dependent as well as time dependent manner. In case of resveratrol, it functions by increasing the mitochondrial membrane potential to breakdown and release cytochrome *c* that ultimately induces apoptosis by several pathway proteins^23–26^. Of particular interest, curcumin, an active ingredient of dietary spice turmeric has gained tremendous attention due to its various pharmacological properties (antioxidant, anti-inflammatory, anti-tumor and anticancer activity). However, the benefits of curcumin are hindered due to its poor water solubility, rapid liver metabolism and limited systemic circulation. Thus, various nanoformulations have been prepared to address the cited problems^27^.

In the current study, a less investigated polymer called Poly-glycerol-malic acid- dodecanedioic acid (PGMD) was used for the preparation of formulations. The polymer was synthesized by Lei et al*.* with slight modifications to the protocol as proposed by Migneco et al*.*^28^. The synthesis was done using the thermal condensation method by mixing glycol, malic acid and 1, 12-dodecanedioic acid (DDA). The polymer was non-toxic due to its naturally occurring by-products such as glycol, DDA and malic acid. Also, the glass transition temperature (Tg) and hydrophilicity can be adjusted by changing the ratio of malic acid and DDA during the synthesis procedure^29^. Further, the MW was observed to be 3000 Da as measured by GPC column and Evaporative Light Scattering Detector (ELSD)^30^. Thus, nanoformulations using hydrophobic or hydrophilic drugs can be successfully developed using the polymer. PGMD nanoparticles have already been implemented for the delivery of imaging agents and drugs such as IR820 and doxorubicin^30, 31^. The PGMD nanoparticles loaded with drug showed good efficacy against MES-SA and Dx-5 cell lines. Also, the nanoformulations showed an improved half-life of IR820 in the mice. Further, studies with IR820/PGMD nanoparticles showed higher expression of HIF-1 and VEGF gene upon treatment with nanoparticles for a longer duration^30, 31^.

Herein, we aimed at synthesis of nanoparticles from PGMD entrapping curcumin using both the variants i.e. 7:3 and 6:4. The nanoparticles were first characterized using Zetasizer for its size and zeta potential. Further, the samples were subjected to FTIR. The efficiency of the synthesized nanoparticles were compared on luminal A and triple-negative breast cancer cell lines (TNBC) (MCF-7 and MDA-MB-231). The efficacy was determined by performing MTT assay on treated cells and IC_50_ was calculated. Along with this, other studies including scratch assay, acridine orange/EtBr, DAPI staining assays and western blot analysis were performed to prove the induction of apoptosis upon treatment with nanoformulations. In addition, the influence of nanoparticles was also studied at the molecular level by evaluation of the expression profile of caspase 9 protein, which is one of the markers of apoptosis in cancer cells. Also, it is worth mentioning here that this is the first-ever study which explores PGMD/CUR nanoparticles for the treatment of breast cancer.

## Material and methods

### Drugs and chemicals

Malic acid, 1, 12-Dodecanedioic acid (DDA), Pluronic 127 were purchased from Sigma. Curcumin and Glycerol were purchased from TCI and Himedia, respectively. Primary (caspase-9 and GAPDH) and secondary antibodies were procured from Santa Cruz, US. Other chemicals used in animal cell culture studies, such as Dulbecco’s modified Eagle’s medium (DMEM), Fetal bovine serum (FBS), Trypsin-EDTA were purchased from Gibco, Thermo Fisher Scientific, India. Phosphate buffer saline, Acridine orange, Ethidium bromide and DAPI were obtained from Himedia, India. MCF-7 and MDA-MB-231 cell lines were procured from NCCS, Pune, India.

### Synthesis of polymer and nanoparticles

The PGMD polymers were synthesized with slight modifications of the protocol as suggested by Migneco et al*.*^28^. Two variants of the polymer were synthesized by changing the molar ratio of DDA and malic acid (7:3 and 6:4). Briefly, DDA and malic acid were mixed at definite ratios in the presence of glycerol and heated up-to 120 °C for 48 h^29^.

**Figure 1:**
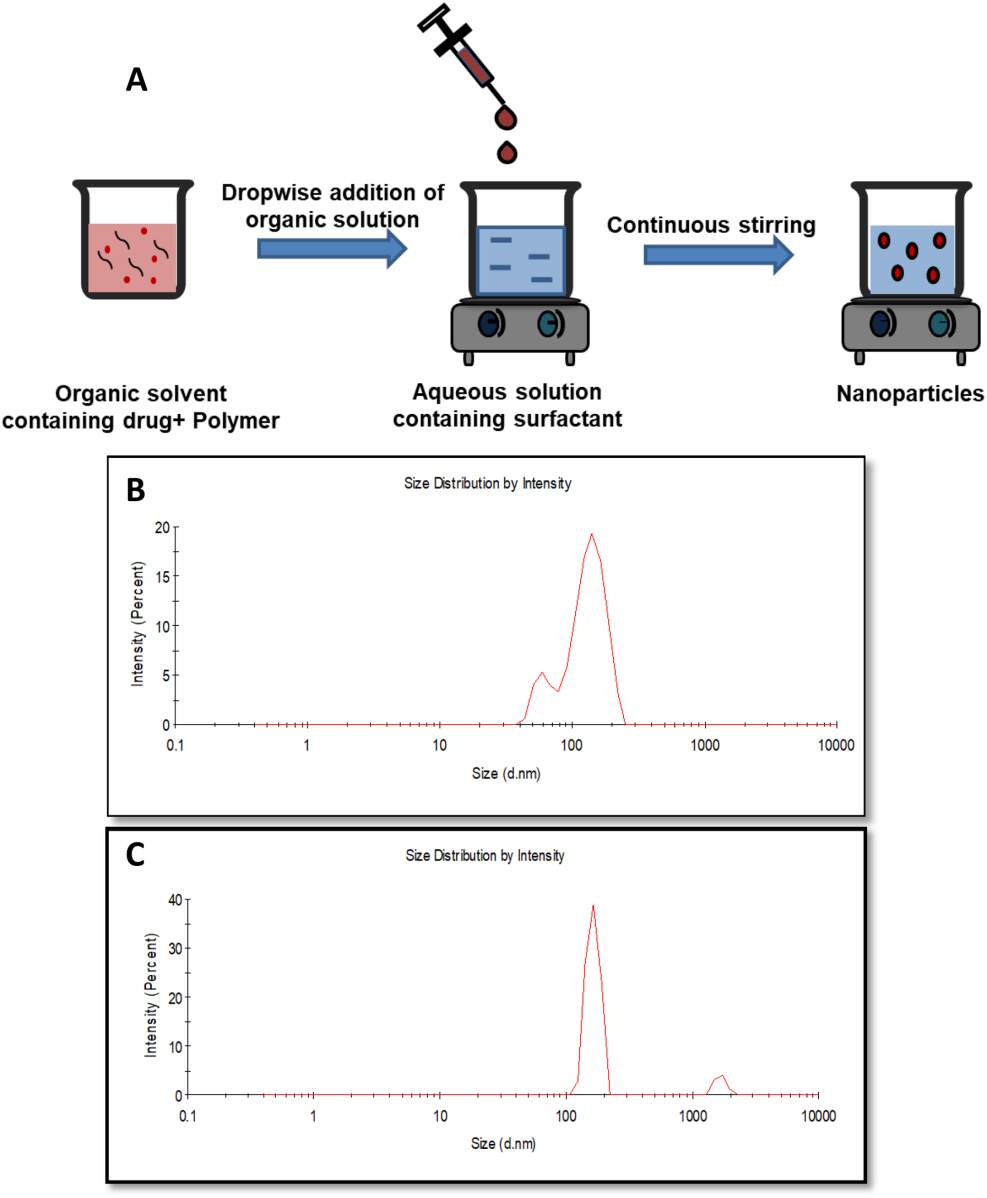
(**A**) Schematic illustration of nanoparticle synthesis by nanoprecipitation method; representative DLS spectra as obtained from zetasizer during size analysis, (**B**) CUR NP 7:3, (**C**) CUR NP 6:4.

The synthesis of PGMD-CUR nanoparticles was done using the nanoprecipitation method. Nanoprecipitation method, also known as solvent displacement method involves precipitation of the polymer from organic to aqueous medium, generally in the presence of surfactant^32^. Further, this technique is considered to be cheap, easy, less complex, and less energy- consuming. However, the presence of a stabilizer such as poloxamer 127 is necessary in order to avoid aggregation during nanoparticle synthesis. Poloxamers, particularly, PF-127 is a non-toxic hydrophilic copolymer which is widely employed in pharmaceutical excipients due to its stabilizing properties and increased solubilization of drugs^33^. Briefly, 0.5 mg of the drug (curcumin) was dissolved in 0.5 mL acetone containing 5 mg polymer (PGMD) (Fig. 1A). The mixed solution was added dropwise to 5 mL 0.1% pluronic solution under stirring at 900 rpm (Stuart hotplate stirrer, UC152, Biocote). The organic phase was removed by leaving it under stirring for 24 h at room temperature. The nanoparticles were then collected by centrifugation at 16,000*g* for 30 min (Heraeus, Fresco 17, Thermo Scientific). Thereafter, it was washed twice followed by lyophilisation for further studies.

### Characterization of nanoparticles

Synthesis of nanoparticles was accomplished as mentioned in the previous section. Additionally, the nanoparticles were centrifuged at 2400*g* that allowed separation of bigger sized particles from the smaller sized particles. The supernatant was collected and further centrifuged at 16,000*g* for 30 min. The pellet thus obtained was washed twice and finally re-suspended in double-distilled water. The size and zeta potential of the samples were analysed using Zetasizer Nano ZS (Malvern, UK) by dispersing the nanoparticles in double-distilled water. Further, nanoparticles were subjected to FTIR spectroscopy to determine potential interaction between the drug and the polymer. Free curcumin and lyophilized nanoparticles were mixed with potassium bromide and analysed in spectrometer from the range 4500–450 cm^−1^.

### Curcumin encapsulation efficiency

The encapsulation efficiency (EE) of curcumin entrapped in the nanoparticles (PGMD/CUR) was determined. This was done by calculating the difference between the total amounts of curcumin used, to the amount of curcumin present in the nanoparticles by dissolving it in DMSO (1 mL). Thereafter, the amount of curcumin was determined by measurement of absorbance on UV–Vis spectrophotometer (Evolution 201, Thermo Scientific). The concentrations of the samples were determined by plotting the standard calibration curve of curcumin in DMSO at 425 nm. The equation used for the calculation was:

$$\% EE = \frac{{\left[ {Total \;amount \;of\; curcumin - Free \;curcumin} \right]}}{Total\; amount \;of \;curcumin} \times 100$$

### In vitro release study

In vitro release study of curcumin loaded onto nanoparticles was performed by investigating its release in PBS solution. Briefly, 5 mg of nanoparticles were suspended in 1 mL PBS (pH 7.4 and pH 5.5) and incubated at 37 °C under shaking condition (50 rpm). The sample containing tubes were taken out at regular time intervals and centrifuged at 16,000*g* for 20 min (Thermo Scientific, Hereus Fresco 17). 0.5 mL of supernatant was then collected and analysed on UV–Vis spectrophotometer. The sample in polypropylene tubes was then replaced by 0.5 mL of fresh PBS and kept in an incubator.

### Cell-viability assay

Human breast cancer cell lines were procured from NCCS, Pune, India. The cells were cultured in high-glucose DMEM media supplemented with 10% FBS, streptomycin (100 μg/mL), and penicillin (100 U/mL) under a humidified atmosphere with 5% CO_2_ at 37 °C. The viability assay was performed by using MTT reagent. Briefly, MCF-7 and MDA-MB 231 cells were plated in 96 well plates at a density of 5 × 10^3^ cells per well and allowed to adhere for 24 h. The cells were then incubated with different concentrations of curcumin nanoparticles with an equivalent amount of curcumin alone. The concentrations were kept in the range from 20–80 µM. The cells were incubated in the CO_2_ incubator at 37 °C at 5% CO_2_ for 24 h and 48 h. After incubation for specified time, 10% MTT (5 mg/mL) was added to each well and incubated for 4 h. Post incubation, the formed formazan crystals were dissolved in DMSO and absorbance was recorded at 570 nm and 620 nm using an Elisa plate reader (Thermo Scientific, Multiskan GO). All the experiments were performed in triplicate using both the variants of the polymer.

### Acridine orange/EtBr and DAPI staining for apoptosis

Acridine orange, a fluorescent dye is taken up by both viable as well as non-viable cells. It intercalates with both double-stranded as well as single-stranded DNA giving a green fluorescence. It is seen that viable cells stains uniformly, whereas cells in early apoptotic stage stains bright green colour. Further, late apoptotic cells stains orange to red in colour, in the presence of EtBr. MCF-7 and MDA-MB 231 cells were seeded in 24 well plates, incubated for 24 h for adherence followed by incubation with or without nanoparticles at a concentration of 40 µM for 24 h. One microliter of dye mixture (100 µg/mL of each acridine orange and EtBr) was mixed with 20 µL of cell suspension. The labelled cells were observed under the fluorescent microscope at 40 × magnification (Leica, DMI 6000B microscope).

For DAPI staining, MCF-7 and MDA-MB 231 cells were treated with nanoparticles as mentioned in the previous paragraph followed washing with PBS and fixation with 4% formaldehyde for 15 min. Cells were then stained using DAPI at a concentration of 3 nM for 10 min and observed under the fluorescence microscope for alterations in the nuclei.

### Scratch assay

The cells MCF-7 and MDA-MB 231 were seeded in a 6-well plate and allowed to reach 70–80% confluency. A scratch was created using a 200 μL pipette tip across the width of the well. Thereafter, the wells were treated with different concentrations of nanoparticles and curcumin alone. The cells were then incubated for 24 h and 48 h, respectively. The cells migration was measured and photographed using an inverted microscope (Zeiss, Aviovert 200) at 0 h, 24 h and 48 h.

### Western blot analysis

MDA-MB-231 cells were seeded in 6-well plate at a cell density of 2 × 10^5^. The cells were treated with 40 μM of curcumin nanoformulations (CUR 7:3 NPs and CUR 6:4 NPs) and curcumin alone the next day. After 48 h of treatment, the cells were lysed using lysis buffer (10 mM Tris, 150 mM NaCl, 0.5 mM and 1% triton X-100 and 1% SDS) incorporated with 1 × complete protease inhibitor cocktail (Thermo Scientific). The lysate was kept on ice and centrifuged at 16,000*g* for 20 min at 4 °C. Thereafter, the supernatants were collected and the protein concentration was determined by Bradford reagent (Himedia). The protein samples were separated by 8% Tris–glycine SDS-PAGE gel by loading 20 μg samples. The gels were transferred overnight onto nitrocellulose membrane (NC) (Biorad) and were blocked for 1 h at room temperature in TBST (50 mM Tris–HCl, pH 7.5, 150 mM NaCl, 0.1% Tween 20) containing 5% non-fat dry milk. The NC membranes were then incubated overnight at 4 °C in 5% milk/TBST with human caspase 9 (mouse, Santa Cruz; 1:600) and human GAPDH antibody (mouse, Santa Cruz; 1:2500) for normalization. Followed by incubation with secondary antibodies coupled to horseradish peroxidase (HRP) (Santa Cruz) at 1:5000 in 5% milk/TBST, immunoreactive bands were revealed by Chemiluminescence using the enhanced chemiluminescence (ECL) Plus kit (Sigma). The Chemiluminescence of the bands on the NC membrane was scanned using Licor. Finally, quantification was performed with Image J software.

### Statistical analysis

All the results were expressed as mean ± standard deviations for triplicate experiments. The mean values obtained in the treatment and control groups were compared and significant differences were determined by two-way ANOVA. Here, p < 0.05 was considered as statistically significant. All the statistical analysis was done using GraphPad Prism 6 (GraphPad Software, San Diego, California).

## Results and discussion

The main focus of the study was to synthesize and characterize PGMD nanoparticles entrapping curcumin to increase its bioavailability to the breast cancer cell lines. Throughout the study, both the variants of polymer i.e. PGMD 7:3 and PGMD 6:4, were used for the synthesis of nanoparticles and treatment of the cancer cells. Migneco et al*.* first synthesized the novel polyester poly (glycerol-dodecanoate) (PGD) from glycerol and dodecanedioic acid (DDA) through ester bonds. It was reported to have good mechanical strength and biological properties^28^. Further, the degradation occurs by hydrolysis mechanism giving rise to products: glycerol and DDA, which are biodegradable and biocompatible^34, 35^. However, PGD is very hydrophobic and has low glass transition temperature (Tg) (32 °C), which makes it a non-ideal candidate for nanoformulations and drug delivery. The biological property that includes biocompatibility and biodegradability of the polymer was modified by adding malic acid at a definite ratio. Thus, for controllable Tg and hydrophilicity of the polymer, the ratio was varied to 7:3 and 6:4 for DDA and malic acid, respectively^29, 30^. Incorporation of DDA and malic acid dictates the hydrophobicity and hydrophilic nature of the polymer. The MW was observed to be 3000 Da as measured by GPC column and Evaporative Light Scattering Detector (ELSD) and reporter in one of our earlier publications^30^. The FT-IR studies of PGMD 7:3 polymer showed the presence of a C=O stretch at 17,333.59 cm^−1^, which is a typical of ester bonds (Figure S1). Further, the characterization of the synthesized polymer onto ^1^H NMR spectra (500 MHz, DMSO-d_6_) showed the relevant chemical shift values pertaining to the structure of PGMD, δ 1.2–1.4 (8 × –CH_2_), 2.2–2.35 (3 × –CH_2_CO), 3.2–3.6 (–OCH_2_, –OCH), 3.8–4.1 (–COCH(OH)–, –OH) (Figure S2).

### Characterization of nanoparticles

It is well known that small particle size has a profound effect in the treatment and diagnosis of several diseases owing to their enormous surface area, high drug loading capacity, controlled drug release and biocompatibility. Nanoparticles of size less than 200 nm persists longer in blood circulation and tend to accumulate in the tumour regions due to the greater size of the pores in the blood vessels in the tumour regions. The porosity develops enhanced permeation effect (EPR) due to more requirements of nutrition in tumour cells. Further, with impaired functioning of the lymphatic drainage system, the EPR effect is much favoured for the nanoformulations^36, 37^. In this study, nanoparticles of size ranging from 110–218 nm were obtained (Fig. 1A). The size of nanoparticles entrapping curcumin synthesized from PGMD, 7:3 (CUR NP 7:3) was ~ 110 nm (Fig. 1B) whereas that from PGMD, 6:4 (CUR NP 6:4) was ~ 218 nm (Fig. 1C). Thus, it can be speculated that these nanoparticles owing to their size smaller or around 200 nm can facilitate uptake by the cancer cells. All the nanoformulations showed size within a desirable range in a monodispersed state with a polydispersity index ranging from 0.17–0.3. In one of the study, Xie et al*.* reported synthesis of curcumin loaded PLGA nanoparticles (CUR-PLGA-NPs) having average size of 200 nm employing solvent evaporation methodology^38^. While varied size PLGA nanoparticles were reported, where PVA and poly (l-lysine) (PLL) were used as stabilizers employing nano-precipitation strategy^39^. The size of nanoparticles entrapping curcumin drastically decreased from 560.4 to 95.6 nm upon increasing the concentration of PVA from 0 to 1% (w/v); and further to 76.2 nm when PLL was employed.

In addition, zeta potential also plays an important role in drug permeation and stability. The zeta potential of CUR NP 7:3 and CUR NP 6:4 were found to be − 18.9 and − 17.5 mV, respectively, which depicts more stability and less aggregation of the particles. Further, the negative charge on the surface of particles was due to the presence of carboxylic groups^40^. In addition, negative zeta potential is more favouring for the EPR effect along with evasion from identification by the phagocytic system, i.e. macrophages and immune system due to low binding of proteins on the surface of the particles^41, 42^. Yallapu et al*.* also reported zeta potential values between − 0.56 and 0.06 mV of curcumin entrapping PLGA nanoparticles synthesized using PVA and PLL as stabilizers^39^. The nanoformulations were speculated to be stable owing to the availability of freely bound PVA/PLL polymer chains onto the surface of nanoparticles. In another study, the zeta potential of curcumin loaded folate-modified-chitosan-nanoparticles was observed to decrease from − 2.32 to − 1.64 mV and from − 6.55 to − 4.25 mV for High MW chitosan and Low MW chitosan, upon substitution of folate^43^.

**Figure 2:**
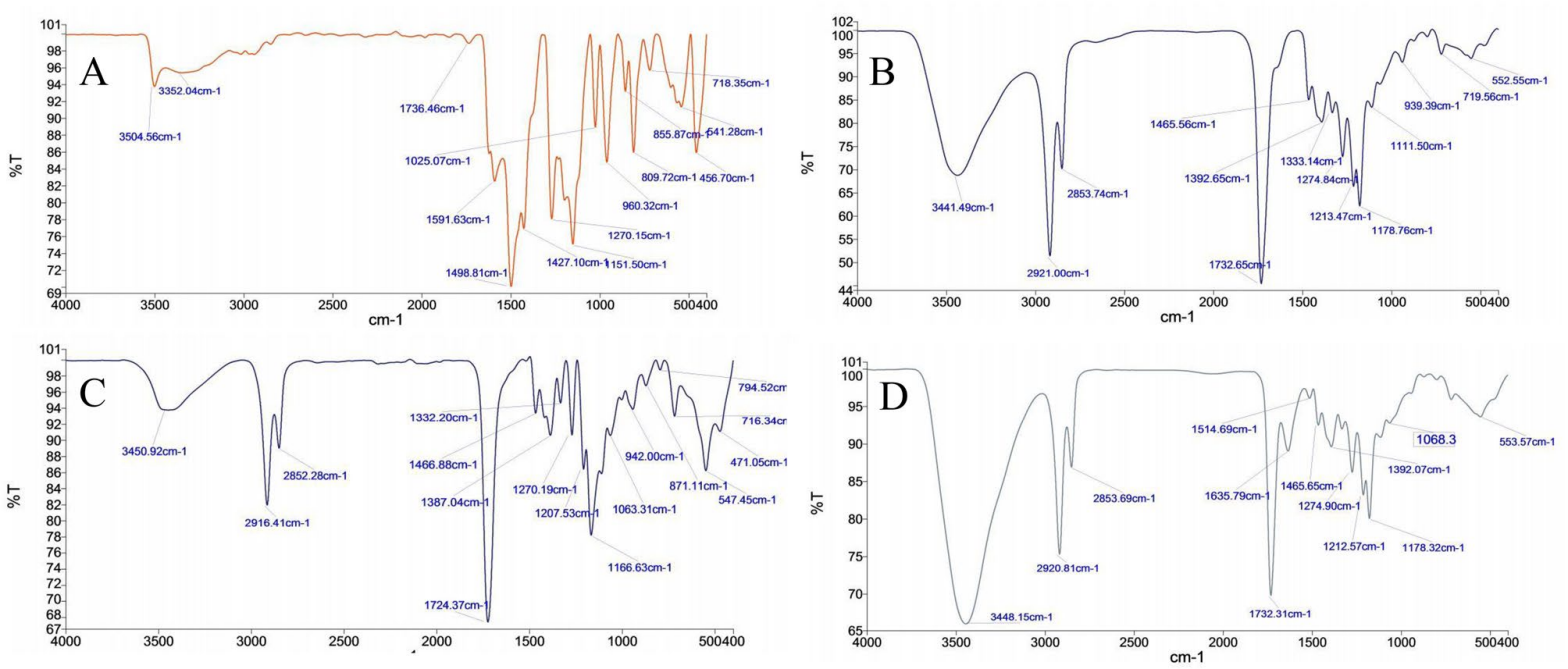
FTIR spectrum of, (**A**) curcumin, (**B**) PGMD polymer, (**C**) 7:3 CUR NPs, (**D**) 6:4 CUR NPs.

As visible from the FTIR spectra of CUR NP 7:3 and CUR NP 6:4, curcumin is depicted by prominent peaks at 3504, 1270, 960, 809.72, 855 and 718.35 cm^−1^ (Fig. 2). The peaks in the range of 718, 809 and 960 cm^−1^ show the bending vibrations of -CH bond of alkene group^44^. The stretching region of the hydroxyl group was seen at the band range of 3200–3600 cm^−1^. The band of carbonyl groups appeared at the region 1736.46 cm^−1^. Similarly, the characteristic peak of PGMD polymer is seen by the presence of a sharp peak around 1732 cm^−1^ in all the CUR NP formulations as well as void nanoparticles. Not much difference was observed in the absorption peaks of void nanoparticles synthesized from both the variants of PGMD polymer. However, there was a slight difference in the absorption peaks of CUR NP 7:3 and CUR NP 6:4. In the case of CUR NP 6:4 a prominent peak at 1635 cm^−1^ indicates the presence of C=C bond indicating the presence of aromatic ring with incorporated curcumin. Whereas, the spectra of CUR NP 7:3 showed slightly different spectra than CUR NP 6:4 with peak shifts in the range of 1500–500 cm^−1^.

### Encapsulation efficiency

Encapsulation efficiency is also an important factor when it comes to anticancer drug delivery. Table 1 shows the EE of curcumin in CUR NP 7:3 and CUR NP 6:4, from which it can be inferred that the EE achieved was nearly the same (~ 75–81%) for both the polymers. The EE depends on several parameters such as polymer concentration, type of surfactant, the volume ratio of aqueous and organic phase etc. In this context, few of the governing parameters were optimized. It was observed that upon increasing the concentration of the polymer from 2 to 5 mg/mL (with all other parameters constant, i.e., 200 µg/mL drug concentration and volume ratio of 5:1) the EE increased. This can be attributed to the fact that with an increase in polymer concentration there is an increase in the viscosity and reduces drug diffusion in the polymeric droplets formed^45^. However, it is evident from other studies that with an increase in polymer concentration the size of the particle increases. Thus, it becomes necessary to select an optimum condition where we obtain good EE with smaller particle sizes. For this purpose, a 10:1 ratio of polymer:drug concentration was considered to be optimum with aqueous to organic phase volume ratio of 5:1. The surfactant, PF127 was selected for synthesis reaction over polyvinyl alcohol (PVA) as higher entrapment was observed with the use of PF127 (~ 70%) than PVA (~ 30%). The other advantages of using PF127 are its low toxicity and immunogenicity^46^. Further, it inhibits the activity of the P-glycoprotein (P-gp) efflux pump, thus ultimately sensitizing the cells 2–3 times upon drug treatment. Also, it is known to overcome glutathione/glutathione-*S*-transferase (GSH/GST) detoxification system that plays a key role in multidrug resistance in human breast cancer cells^47, 48^. On the other hand, PVA, though widely used can prove to be carcinogenic^49^. Previous studies show that the surfactant, PVA, cause hypertension, organ lesions, anaemia, and depression of the central nervous system in animals upon subcutaneous and intravenous administration^50^. The reduced entrapment with PVA could be discerned from the explanation of its properties; PVA is a highly hydrophilic stabilizer that is known to reduce nanoparticles stability in aqueous solutions. The inclusion of PVA increases the solubilization rate of polymeric nanoparticles in the aqueous medium resulting in decreased entrapment efficiency and loss of drug^51^. Further, the optimum stirring speed for the reactions was set at 700 rpm.

**Table 1:** Represents the encapsulation efficiency of CUR NP 7:3 and CUR NP 6:4.

Formulations	Polymer (mg/mL)	Drug (mg/mL)	Volume ratio of aqueous and organic phase	Encapsulation efficiency (%)
CUR NP 7:3	10	1	5:1	81.6
CUR NP 6:4	10	1	5:1	76.51

### In vitro release study

**Figure 3:**
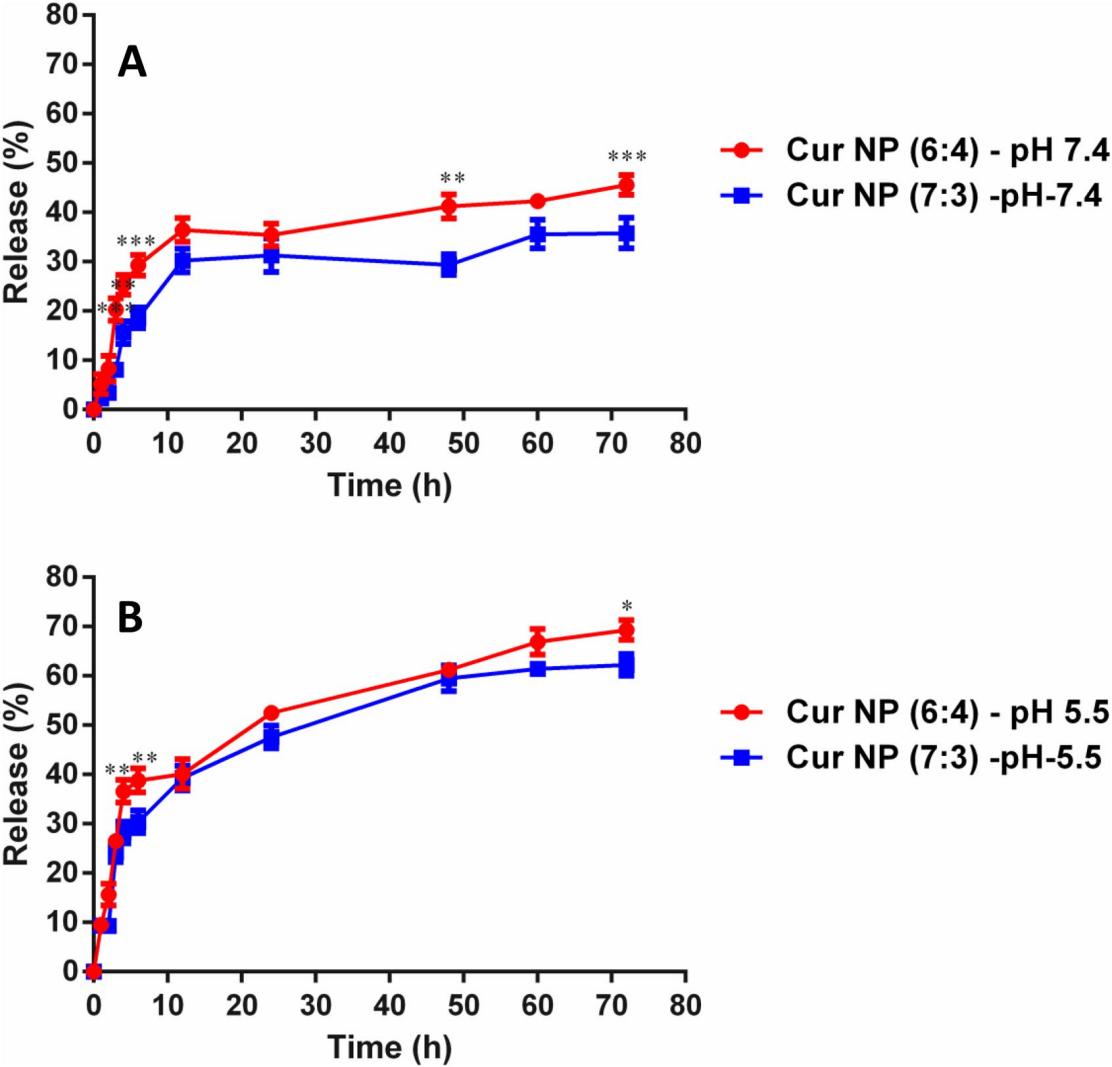
In vitro release study of CUR nanoparticles in PBS, (**A**) at pH 7.4 and (**B**) pH 5.5. Statistical analysis was performed using multiple variance analysis (two way ANOVA) with Bonferroni Test. (*p < 0.05, **p < 0.01, ***p < 0.001 and n = 3).

The release profile of the drug was investigated in PBS solution at pH 7.4 and 5.5. It was observed that the release profile of the drug from the nanoformulations were slow. In pH 7.4 the maximum release reached to 35.8% for CUR NP 7:3, whereas, in the case of CUR NP 6:4, the release of the drug was found to be in the range of 45.6% till 72 h of time duration (Fig. 3A). Contrastingly, the curcumin release profile was found to be significantly higher at pH 5.5 relative to pH 7.4. The release of drug enhanced to 62.2% for CUR NP 7:3, whereas in the case of CUR NP 6:4 the release reached as high as 69.3% within 72 h (Fig. 3B). The probable reason for higher release in the acidic environment could be due to faster degradation and hydrolysis of the polymer^52^. It is a well-known fact that the tumour environment possesses acidic pH relative to healthy tissues and blood^53^. Thus, accelerated release of curcumin in the tumour could prove promising in cancer therapy. Further, cancer is accompanied by long term treatment and multiple dosing. This affects patient compliance and standard of living. Controlled and extended release of drugs from nanoformulations is a recent attractive therapeutic approach for chronic diseases, especially cancer. This can have a profound effect on the treatment of cancer by reducing multi-dosing and other side effects^54, 55^. Currently, hydrophobic polymeric vectors have been implemented for the delivery of therapeutic molecules to the tumour site. Thus, the slow and controlled release of PGMD/curcumin nanoparticles can prove to be a better alternative for cancer therapy. In one of our previous studies, doxorubicin (DOX) was entrapped in PGMD nanoparticles and in vitro release was monitored for 29 days^30^. At pH 7.4 and pH 5.0, around 49% and 72% of DOX was released in initial 5 h, followed by a slow release that reached a total of only ~ 52% and 86% after 29 days, respectively. A similar kind of pattern was observed for release of curcumin from curcumin–chitosan (CUR–CS) and curcumin–cyclodextrin–chitosan (CUR–CD–CS) nanoconjugates^56^. Both the formulations showed initial burst release of about 10% (up to 12 h) in case of CUR-CS formulation slower release kinetics was observed with only ~ 45% of total CUR released after 72 h. While in case of CUR-CD-CS, much faster release kinetics was shown with up to 70% of total CUR released after 72 h. In another study with curcumin loaded folate-modified-chitosan-nanoparticles, a sustained release done at pH 5 and pH 7.4 for a period of one week showed ~ 90% and 75% of curcumin release^43^.

### Cell viability assay

**Figure 4:**
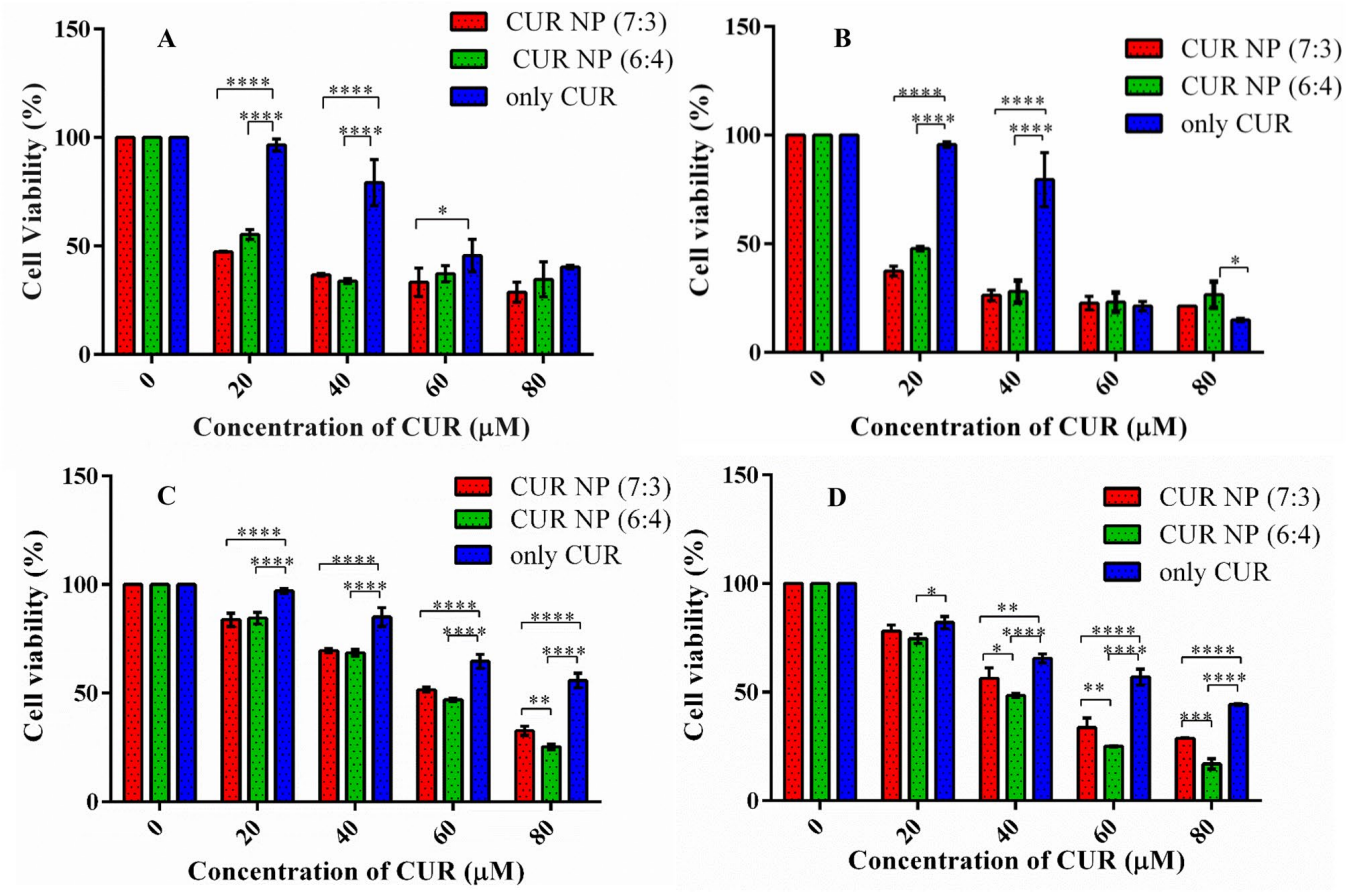
Cell viability as determined by MTT assay. The % cell viability of nanoparticles and curcumin treated cancer cell lines at different time intervals, (**A**) % cell viability of MCF-7 cells after 24 h, (**B**) % cell viability of MCF-7 cells after 48 h, (**C**) % cell viability of MDA-MB 231 cells after 24 h, and (**D**) 48 h. Statistical analysis was performed using multiple variance analysis (two way ANOVA) with Tukey’s test (*p < 0.05, **p < 0.01, ***p < 0.001, ****p < 0.000, *ns* not significant and n = 3).

**Figure 5:**
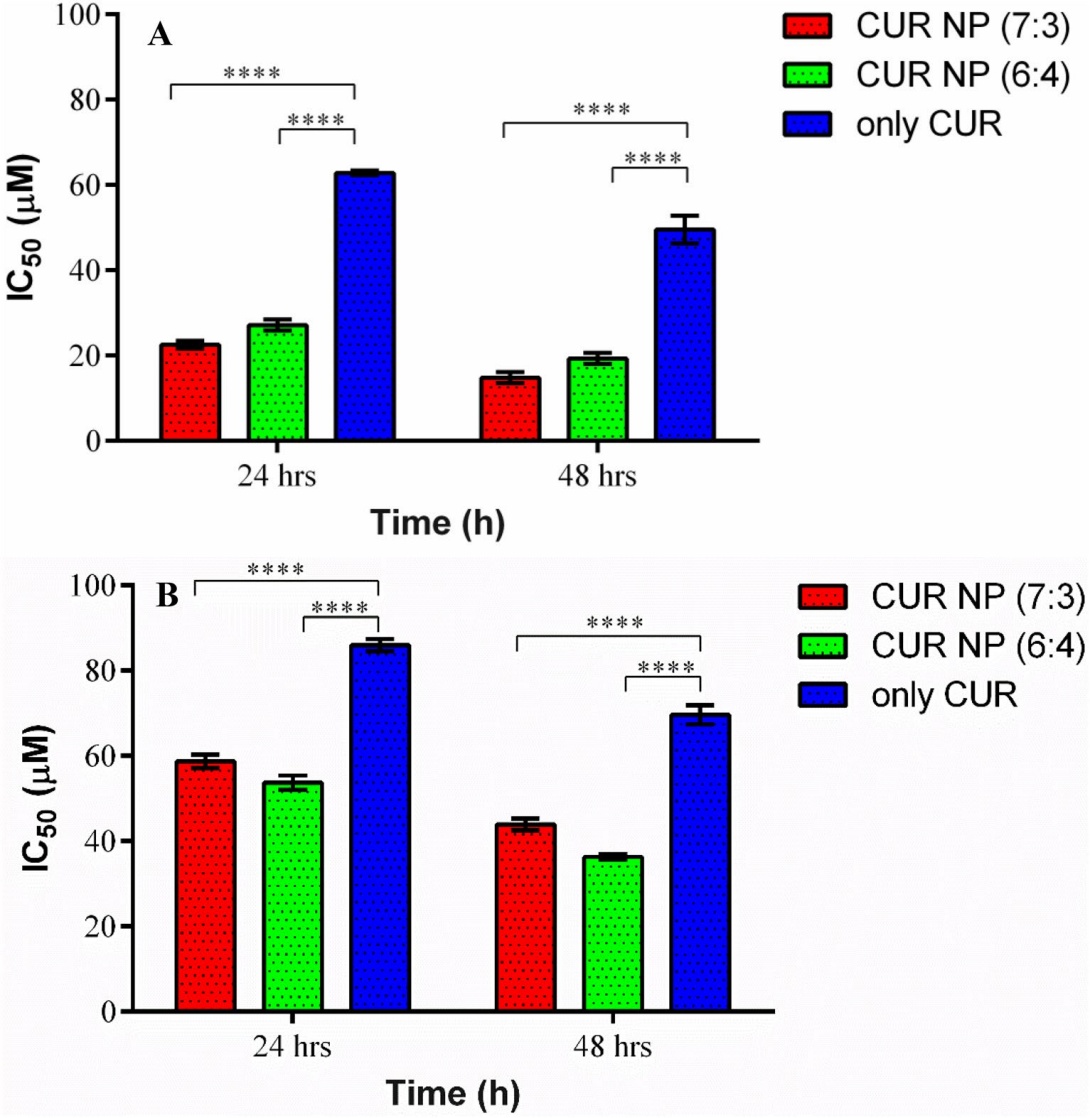
Represents IC_50_ values of CUR NP (7:3), CUR NP (6:4) and CUR only at 24 h and 48 h on (**A**) MCF-7, (**B**) MDA-MB-231 cell line. Statistical analysis was performed using multiple variance analysis (two way ANOVA) with Tukey’s test. (****p < 0.0001, *ns* not significant and n = 3).

**Figure 6:**
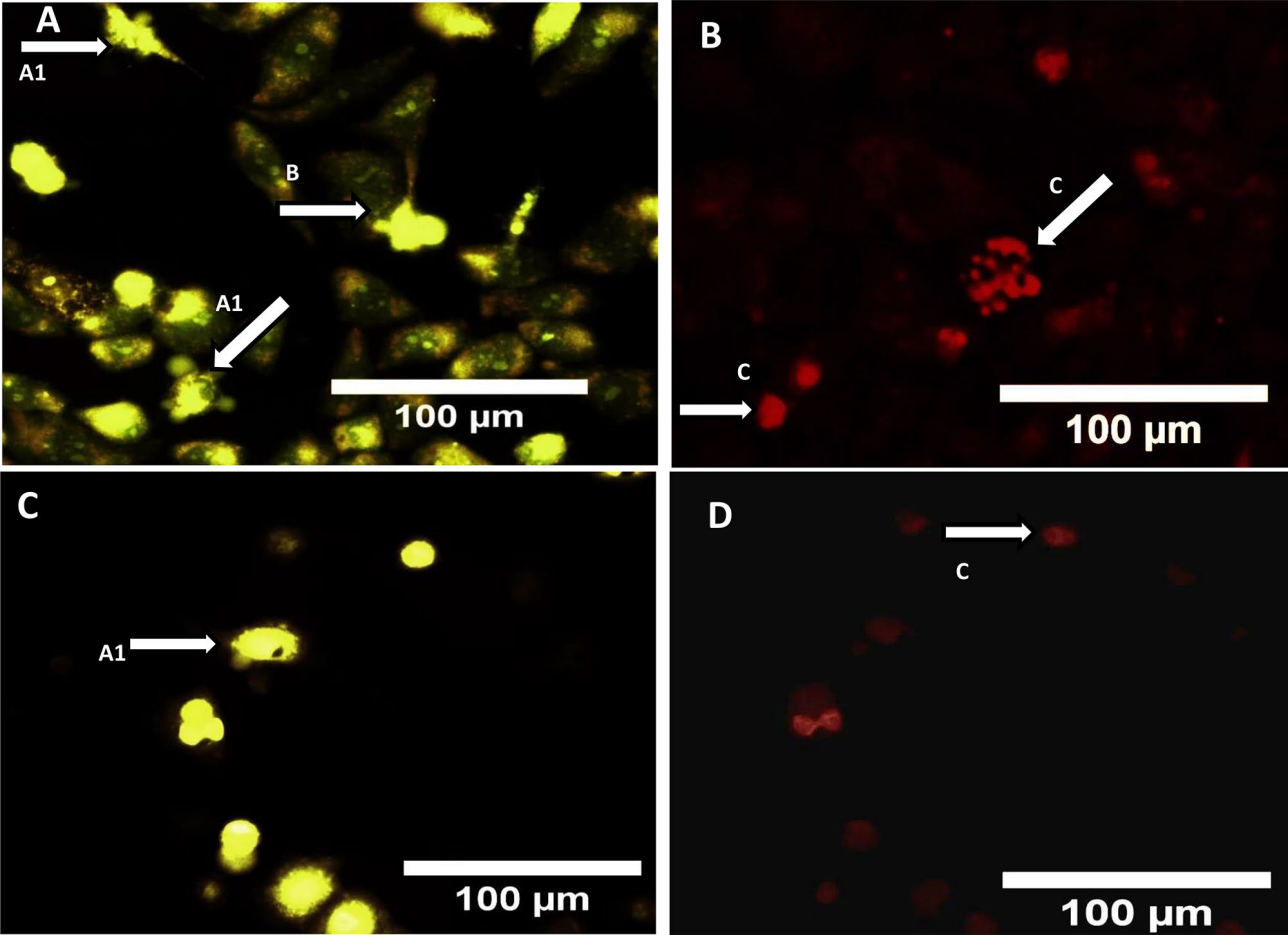
AO/EtBr stained images of (**A**,**B**) MDA-MB-231 cells treated with curcumin nanoparticle (**C**,**D**) represents MCF-7 cells treated with curcumin nanoparticle, where (**A1**) and (**A2**) membrane blebbing, (**B**) early apoptotic cell, (**C**) fragmented nucleus, (**D**) dead cell.

**Figure 7:**
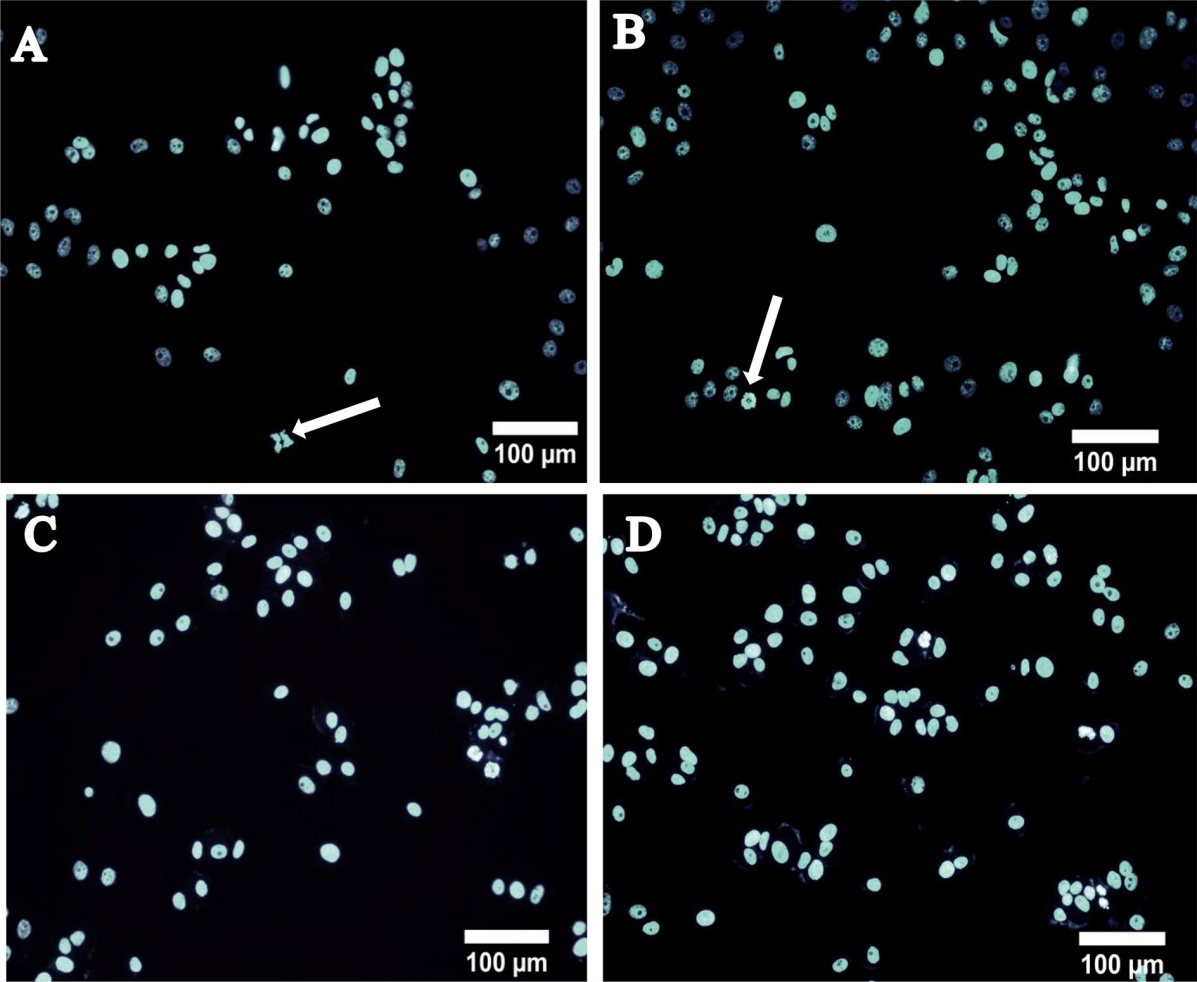
DAPI stained fluorescent images of MCF-7 cells treated at a concentration of 40 µM for 24 h, (**A**) CUR-NPs 7:3, (**B**) CUR-NPs 6:4, (**C**) CUR only, (**D**) control.

Breast cancer accounts for a total of 25% annual global burden of cancer. Despite of wide range of treatment strategies, it has been observed that 6–10% are metastatic with 30% recurrences^57, 58^. Thus, a novel treatment regimen is the need of the hour. For this purpose, curcumin, a hydrophobic polyphenolic compound derived from *Curcuma longa* was selected owing to its wide range of applications such as antifungal, antiviral, antioxidative, anti-inflammatory, and anti-proliferative activities^59–61^. However, the major drawback of the compound is its poor water solubility and bioavailability^62–64^. This drawback could be circumvented by the use of nanoformulations. In this study, the anticancer activity of CUR NPs synthesized using both the polymer variants (PGMD 7:3 and PGMD 6:4) were tested on MDA-MB 231 and MCF-7 cell lines. The cells were exposed to both the CUR-NPs at a varying concentration (20 μM, 40 μM, 60 μM and 80 μM) for 24 h and 48 h. Consecutively, the cells were also treated with curcumin alone at the same concentrations mentioned previously. Results demonstrated curcumin nanoparticles to be more potent than curcumin alone (Fig. 4). Also, the nanoparticles showed a decrease in cell viability in a time and dose-dependent manner. Previous studies have reported that human breast cancer cell lines (MCF-7 and MDA-MB-231) treated with curcumin showed anticancer activity by inducing apoptosis in cells^65^. In another study, myristic acid-chitosan (MA-chitosan) was used for nanoformulation against MDA-MB-231 cells, which showed potent activity relative to curcumin alone with reduced IC_50_ value for nanocurcumin^66^. Similarly, apoptotic morphological changes were observed such as shrinkage, cytoplasmic blebbing and rounding of cells (Figs. 6 and 7). The anticancer activities of both the CUR-NP formulations were nearly the same in MCF-7 cell lines and the IC_50_ of both the formulations (CUR-NP 7:3 and CUR NP 6:4) was found to be in the range of 14.83 ± 1.31 and 19.32 ± 1.31 μM for 48 h (Fig. 5A) respectively. Similarly, in the case of MDA-MB 231 treated cell lines, the decrease in cell viability was seen in a time- dependent manner with an increase in the concentration. The IC_50_ values of CUR-NPs (7:3 and 6:4) on MDA-MB-231 cell lines were found to be in the range of 43.91 ± 1.35 and 36.28 ± 0.63, respectively (Fig. 5B). The IC_50_ values are collectively displayed in Table 2. Our results are in good concurrence with what was observed with curcumin entrapped PLGA nanoparticles study conducted in ovarian (A2780CP) and breast (MDA-MB-231) cancer cells^39^. Both free CUR and nano-CUR showed dose dependent anti-proliferative effects where higher efficacy was seen in nano-CUR. The IC_50_ of nano-CUR6 was observed to be 13.9 and 9.1 μM in A2780CP and MDA-MB-231 cancer cells, respectively. In study reported with curcumin loaded folate-modified-chitosan-nanoparticles, the viability of MCF-7 cells was observed to decrease with the increase in the incubation time^43^. The cytotoxic efficacy of free CUR was observed to decrease after prolonged incubation while of CUR-loaded nanoparticles slightly increased after 7 days.

**Table 2:** Depicts the IC_50_ values of CUR NPs and CUR alone treated on MCF-7 and MDA-MB-231 cell lines.

Nano-formulations	MCF-7		MDA	
	24 h	48 h	24 h	48 h
CUR NP (7:3)	22.54 ± 0.91	14.83 ± 1.31	58.71 ± 1.57	43.91 ± 1.35
CUR NP (6:4)	27.17 ± 1.32	19.32 ± 1.31	53.72 ± 1.67	36.28 ± 0.63
CUR only	62.82 ± 0.57	49.57 ± 3.26	85.93 ± 1.43	69.62 ± 2.24

### Acridine orange/ethidium bromide and DAPI staining

**Figure 8:**
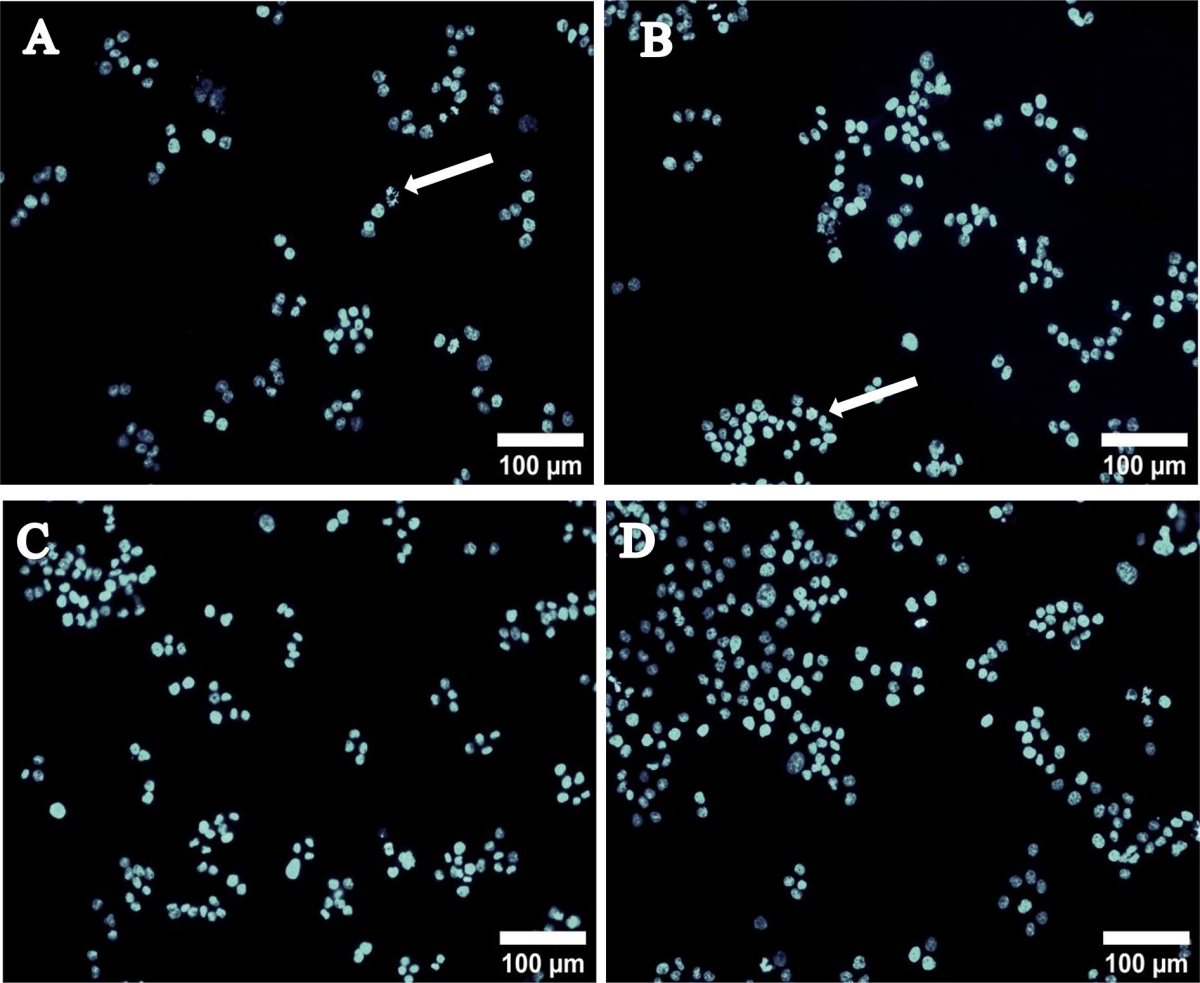
DAPI stained fluorescent images of MDA-MB-231 cells treated at a concentration of 40 µM for 24 h, (**A**) CUR-NPs 7:3, (**B**) CUR-NPs 6:4, (**C**) CUR only, (**D**) control.

The induction of cytotoxicity can be confirmed by performing AO/EtBr staining to assess apoptosis. Apoptosis is a major process that maintains homeostasis of tissues and is considered as an ideal way for inhibiting cancer cell proliferation^67^. AO/EtBr staining is done for morphological assessment of cells undergoing apoptosis after treatment with anticancer drugs or other specific drugs targeting apoptosis. Also, this method has been one of the preferred methods for detecting any nuclear morphological changes microscopically^68–70^. AO penetrates normal as well as early apoptotic cells with an intact membrane and gives green fluorescence. Whereas, EtBr is mainly taken up by cells that have damaged cell membranes, cells that are at late apoptotic or dead cells, emitting orange-red colour when intercalated with DNA or damaged bodies^71^. Thus, from the fluorescent images of MDA-MB-231 and MCF-7 cells (Fig. 6A–D) it is evident that live cells give green fluorescence and early apoptotic cells emit bright green colour with condensed nucleus/chromatin. On the other hand, late apoptotic cells show yellow-orange colouration and necrotic cells as the orange or red uniform nucleus. Thus, in both the cell lines characteristic signs of apoptosis was observed, such as cell shrinkage, the formation of apoptotic bodies and membrane blebbing^72^. Similarly, upon staining cells with DAPI it was obvious from the results, that the treated cells showed chromatin condensation and fragmentation, which could be related to the induction of apoptosis in relative to normal untreated cells (Figs. 7 and 8). Similar results have been reported by Reeves et al*.* where the nanogel formed with curcumin showed greater activity against MDA-MB 231 cells in relative to curcumin alone^73^. Folate coated PEG-PLGA has also been investigated for curcumin entrapment that showed enhanced apoptotic activity on the HeLa cells^74^. Study with curcumin-containing chitosan nanoparticles (CUR-CS-NP) also reported fragmentations of nuclei and appearance of irregular edges upon treatment of HT-29 cells with free curcumin and CUR-CS-NP at doses of 75 μM which clearly suggested of cells undergoing apoptosis^75^.

### Scratch assay

**Figure 9:**
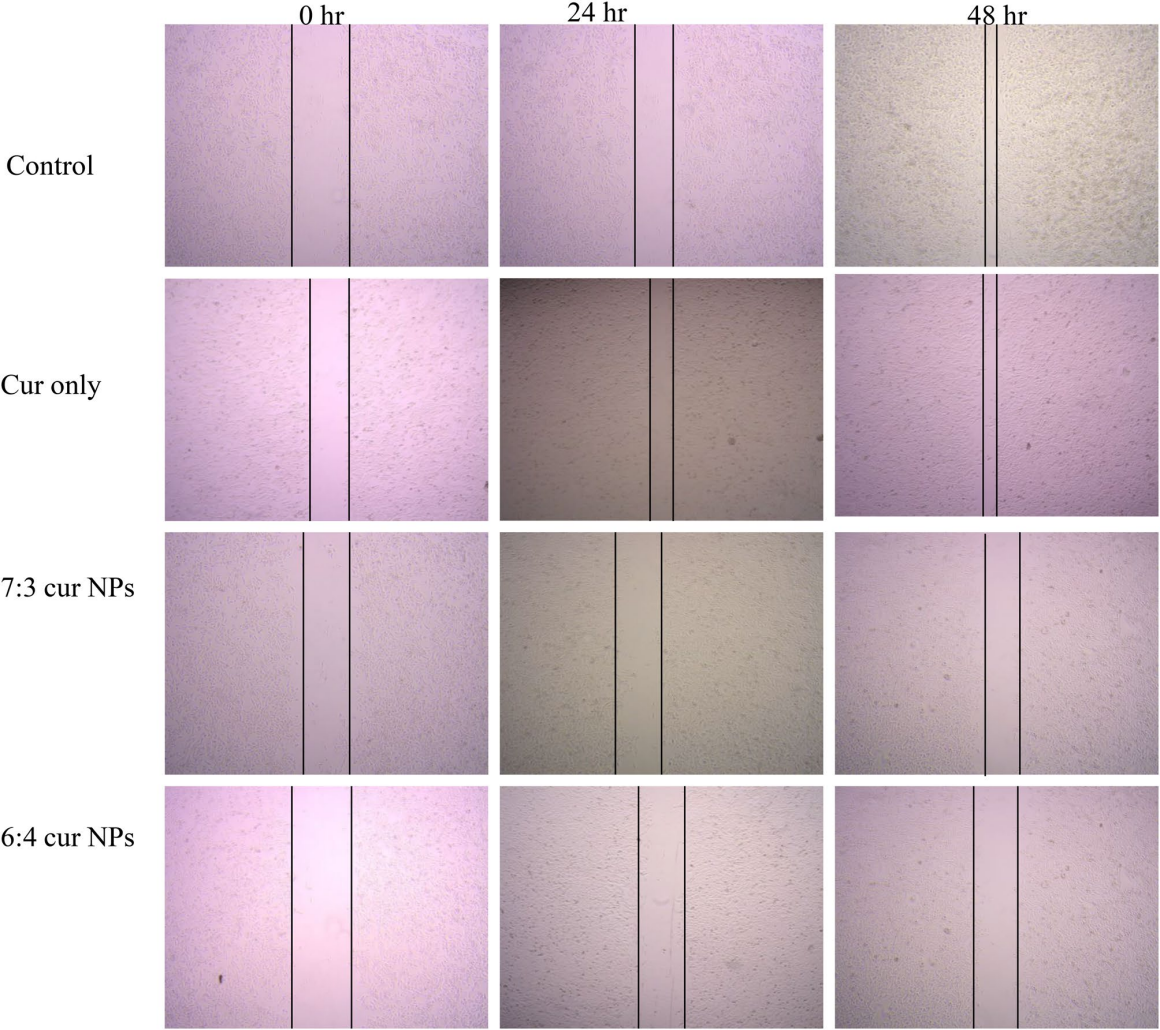
Microscopic images of scratch assay performed on MCF-7 cell line treated with CUR NPs and CUR alone.

**Figure 10:**
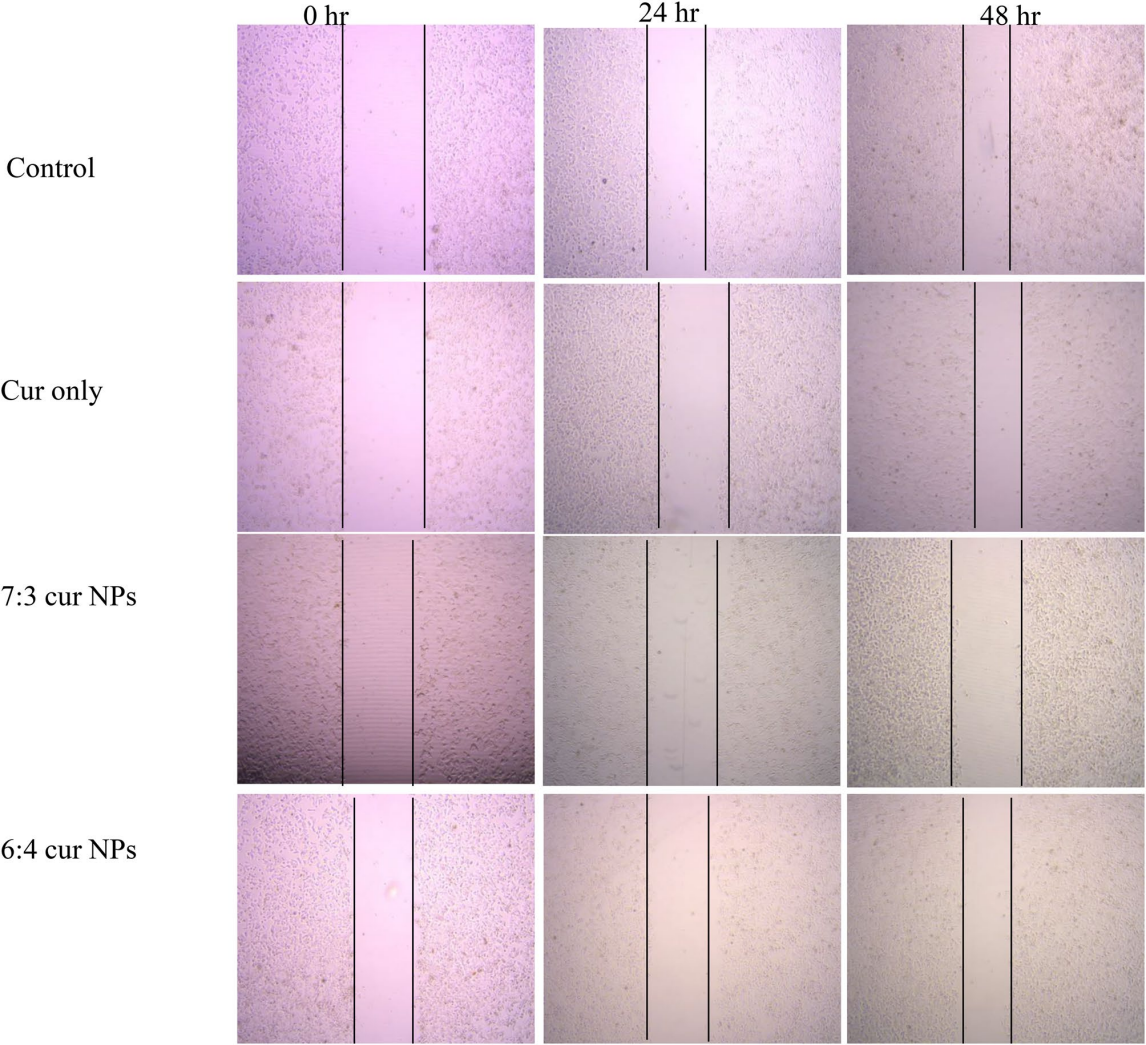
Microscopic images of scratch assay performed on MDA-MB-231 cell line treated with CUR NPs and CUR alone.

**Figure 11:**
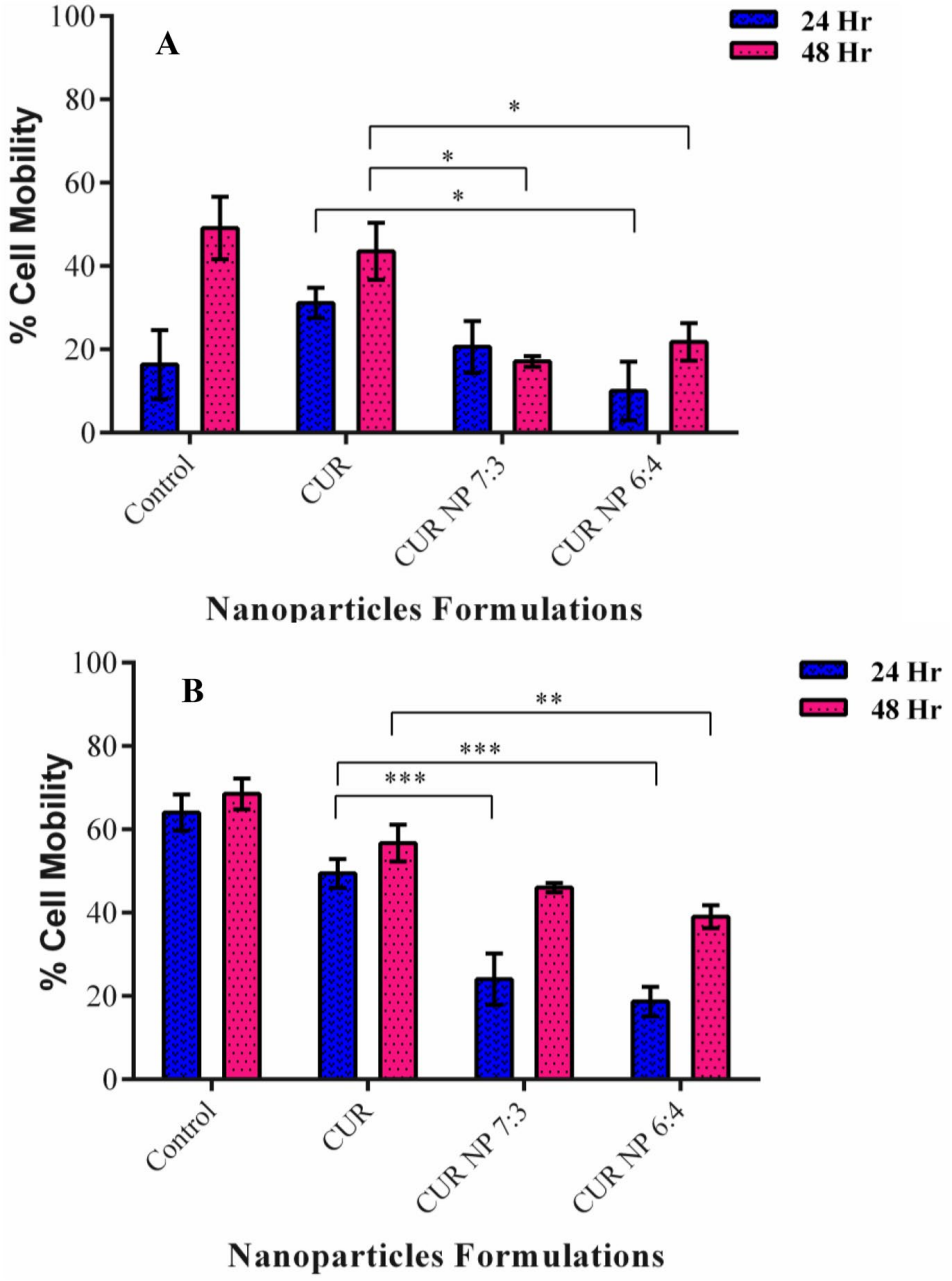
Quantitative representation of scratch assay in (**A**) MCF-7 and (**B**) MDA-MB-231 cell lines. Statistical analysis was performed using multiple variance analysis (two way ANOVA) with Tukey’s test. (*p < 0.05, **p < 0.01, ***p < 0.001, *ns* not significant and n = 3).

Metastasis of cancer cells is the main characteristic of advanced stage cancer as well as reason for the overall mortality in patients with cancer. In this stage, the cells migrate through the basement membrane and vasculature to other organs. Thus, it becomes imperative to design drugs that can inhibit this activity effectively^76, 77^. Scratch assay is a commonly employed method to evaluate the cell migration of cancer cells in-vitro. Further, it is evident from previous studies that curcumin has anti-proliferative and apoptotic activity in breast cancer cells^78, 79^. Upon treatment with CUR NP 7:3 and CUR NP 6:4 it was observed that the wound closure decreased dramatically in comparison to the control cells where the migration of cells was aggressive including the cells treated with curcumin only. The percent cell migration mobility was found to be 44.9%, 43.8%, 17.09% and 21.04% for control, curcumin, CUR 7:3 NP and CUR 6:4 NP treated cells respectively for MCF-7 cells (Fig. 9) after 48 h of treatment. In the case of MDA-MB 231 cells, the percent cell migration mobility was found to be 68.5%, 49.5%, 45.9% and 39.1% for control, curcumin, CUR 7:3 NPs and CUR 6:4 NPs treated cells respectively for 48 h (Fig. 10). It could be noted that the cell migration mobility of MDA-MB 231 cell lines was more in comparison to the MCF-7 cell lines which could be attributed to the triple negative and aggressive nature of MDA-MB 231 cell line (Fig. 11). Study performed with curcumin-loaded galactosylated BSA nanoparticles (Gal-BSA-Cur NPs) reported of lower wound closure as compared to CUR alone or BSA-Cur NPs suggesting of inhibition of migration of HepG2 cells, thereby substantiating the tumor-suppressive role of nanoparticles^80^. Further, the scratch assay performed on PANC-1 and Mia Paca-2 cells treated with curcumin loaded chitosan/PEG blended PLGA nanoparticles showed an effective inhibition in the migratory efficacy of the cells in contrast to treatment with free curcumin^81^.

### Western blot analysis

**Figure 12:**
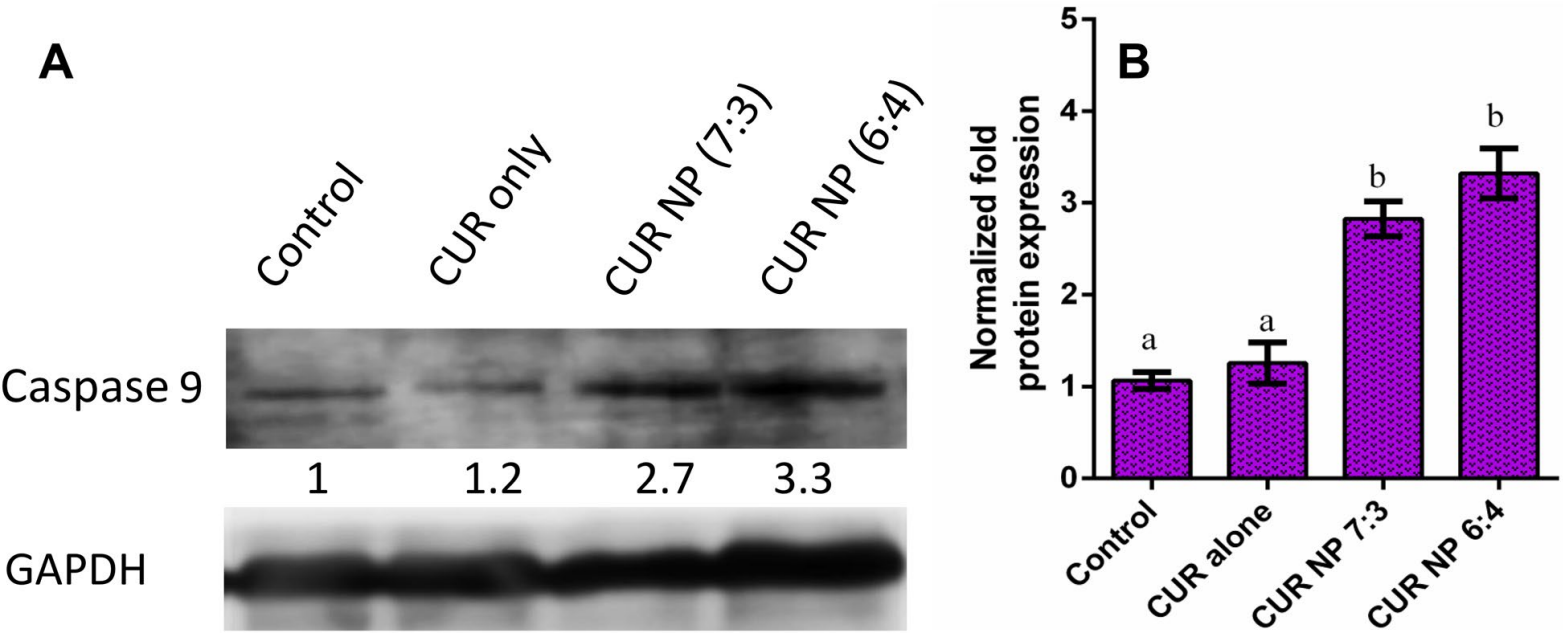
Western blot analysis study, (**A**) MDA-MB-231 cell line treated with nanoparticles showing overexpression of caspase 9 relative to control and cells treated with curcumin alone, (**B**) Normalized protein expression. Statistical analysis was performed using multiple variance analysis (one way ANOVA) with Tukey statistical analysis was performed using multiple variance analysis (one way ANOVA) with n = 3.

The effect of curcumin nanoparticles at the molecular level was determined by evaluating the caspase-9 expression profile. Extensive studies on curcumin have shown its role in modulating signalling molecules associated with cell proliferation as well as apoptosis. Apoptosis occurs through two major pathways i.e. extrinsic and intrinsic pathways. Herein, curcumin can activate apoptosis by changing the membrane potential or reactive oxygen species^82, 83^. In the mitochondrial pathway, curcumin induces the release of cytochrome-*c* and enhances the expression of caspase-3 and caspase-9. Further, PARP causes damage to DNA and apoptosis. In addition, the upregulation of Bad and Bax along with downregulation of Bcl-2 and Bcl-XL also play an important role in curcumin-induced pathway. Also, it is well established that activation of apoptosis pathways is independent of hormone receptors (estrogen receptor (ER), progesterone receptor (PR) and HER) expressed on the breast cancer cells and that the triple-negative cancer cells also exhibit similar effect^84–87^. Thus, in the current study, treatment of MDA-MB-231 cells with both the nanoformulations (CUR NP 7:3 and CUR NP 6:4) displayed overexpression of caspase-9 relative to control untreated cells, and cells treated with curcumin drug alone (Fig. 12). In one of the previous study, Chang et al*.* investigated delivery of curcumin loaded onto PLGA nanoparticles to CAL27-cisplatin-resistant human oral cancer cells (CAR cells) and reported induction of caspase 9 when treated for 24 h^80^. Further, Peng et al*.* also studied curcumin loaded onto PLGA nanoparticles effect onto U2OS human osteosarcoma cells and suggested stimulation of caspase 9 in a time dependent manner which contributes to induction of apoptosis^88^. Our results are in well agreement with these published works.

## Conclusion

In the current study, PGMD/curcumin nanoparticles were successfully synthesized using both the variants of polymer i.e. PGMD 7:3 and PGMD 6:4. Further, both (CUR 7:3 NPs and CUR 6:4 NPs) the nanoparticles showed potential anticancer activity towards breast cancer cell lines (MCF-7 and MDA-MB 231) by MTT assay. Though, the polymer PGMD 6:4 is more hydrophilic than PGMD 7:3; there was no significant difference found between the anticancer activities by both the nanoparticle formulations on breast cancer cell lines. The scratch assay showed its potential against the anti-metastatic activity. Acridine orange/ETBR, DAPI staining and western blot studies also displayed the apoptotic mediated cell death of the cancer cells. Overall, the study showed that the nanoparticles displayed increased anticancer activity than curcumin alone. Nevertheless, more advanced studies are required with further investigation. We strongly believe that the PGMD/CUR nanoparticles synthesized could be used as a potential anticancer agent in the near future.

## Supplementary Information

Supplementary Information 1.

Supplementary Information 2.
