# Supplementary material for: PGMD/curcumin nanoparticles for the treatment of breast cancer
Source: Sci Rep. 2021 Feb 15;11:3824. doi: 10.1038/s41598-021-81701-x (PMC7884397; doi:10.1038/s41598-021-81701-x)
Supplement: Supplementary file 1 — Supplementary Information 1. [file 41598_2021_81701_MOESM1_ESM.pdf]

Supplementary Materials:

Polymer Characterization

For the functional group analysis, vacuum dried samples of PGMD 7:3 was analysed by Fourier transform infrared spectroscopy (FTIR). The pellets was prepared along with KBr and scanned in the range of 4000 to 400 cm^{-1} against blank KBr pellet. The FT-IR studies of PGMD 7:3 polymer showed the presence of a C=O stretch at 17333.59 cm^{-1}, which is a typical of ester bonds.

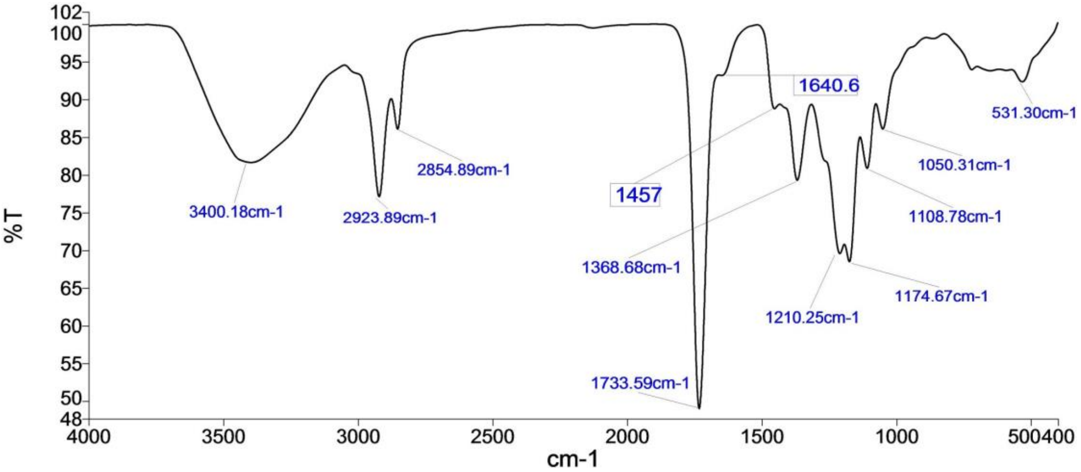

Figure S1 – FTIR Spectrum of PGMD 7:3 polymer

The characterization of the synthesized polymer onto ^{1}H NMR spectra (500 MHz, DMSO-d_6) showed the relevant chemical shift values pertaining to the structure of PGMD, δ 1.2-1.4 (8x –CH_2), 2.2-2.35 (3x –CH_2CO), 3.2-3.6 (-OCH_2, -OCH), 3.8-4.1 (-COCH(OH)-, -OH) (Figure S2).

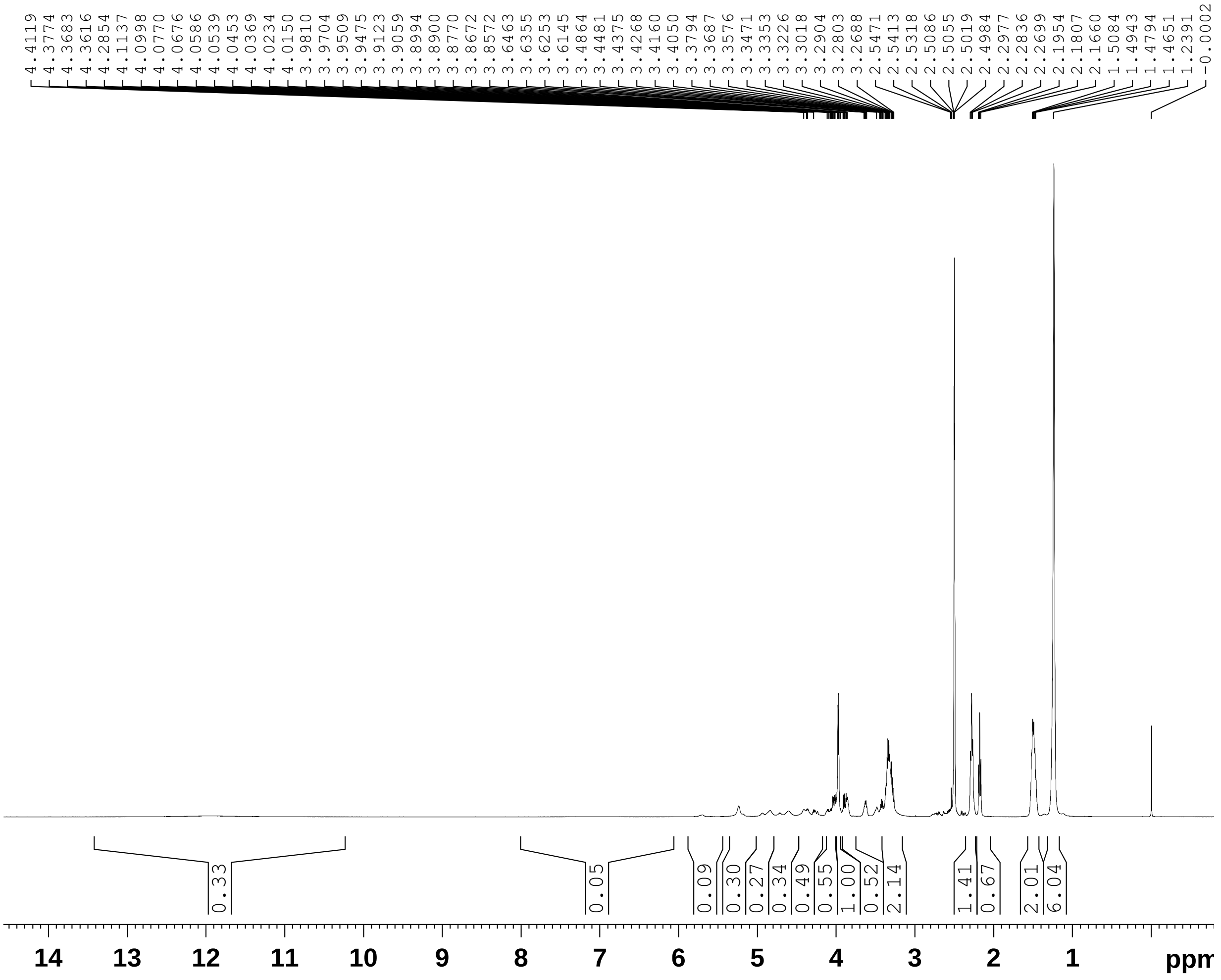

Figure S2 – ^{1}H NMR Spectrum of PGMD 7:3 polymer
