# Supplementary material for: PGMD/curcumin nanoparticles for the treatment of breast cancer
Source: Sci Rep. 2021 Feb 15;11:3824. doi: 10.1038/s41598-021-81701-x (PMC7884397; doi:10.1038/s41598-021-81701-x)
Supplement: Supplementary file 2 — Supplementary Information 2. [file 41598_2021_81701_MOESM2_ESM.pptx]

## Slide 1
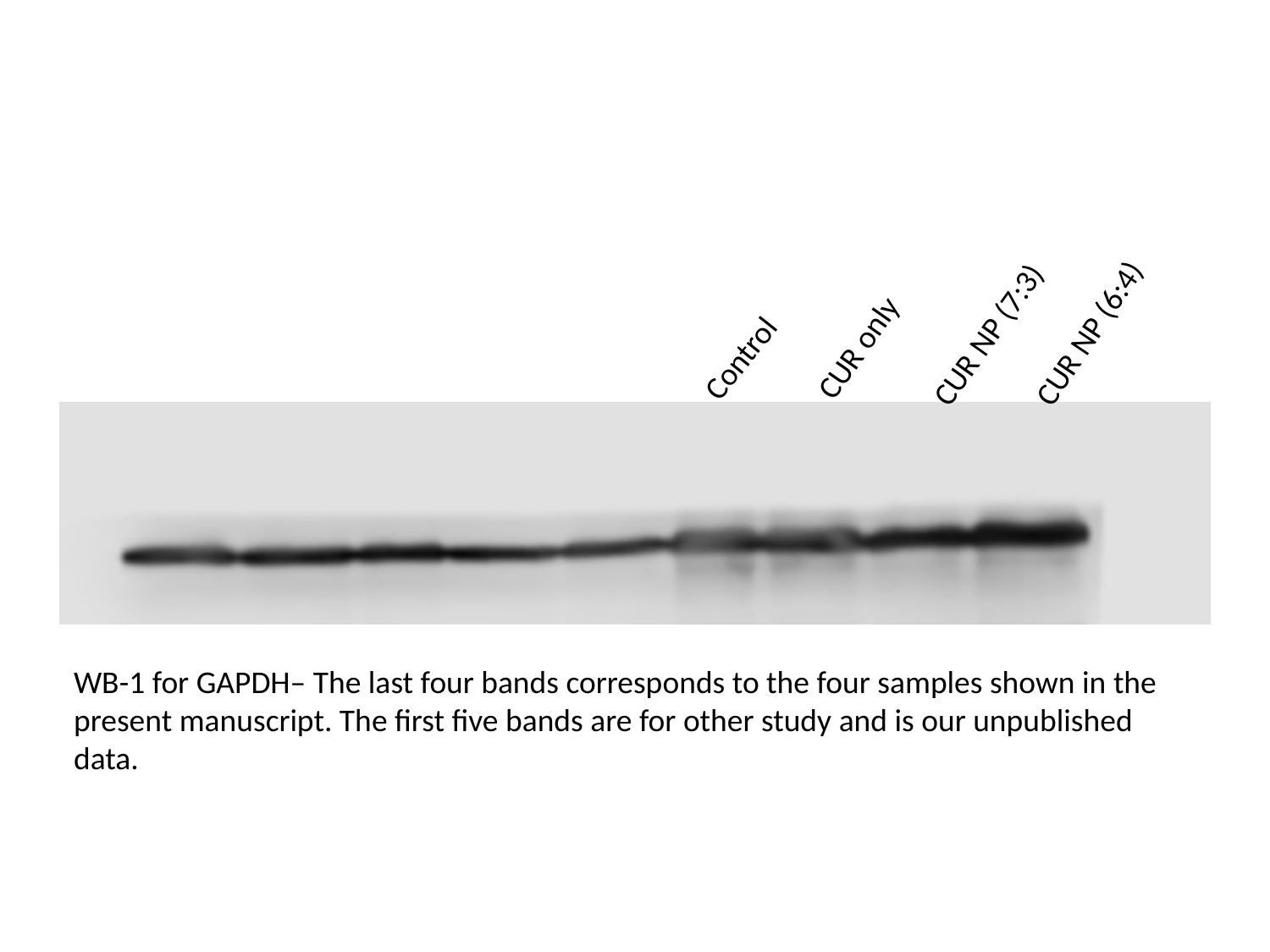

CUR NP (6:4)
CUR NP (7:3)
CUR only
Control
WB-1 for GAPDH– The last four bands corresponds to the four samples shown in the present manuscript. The first five bands are for other study and is our unpublished data.

## Slide 2
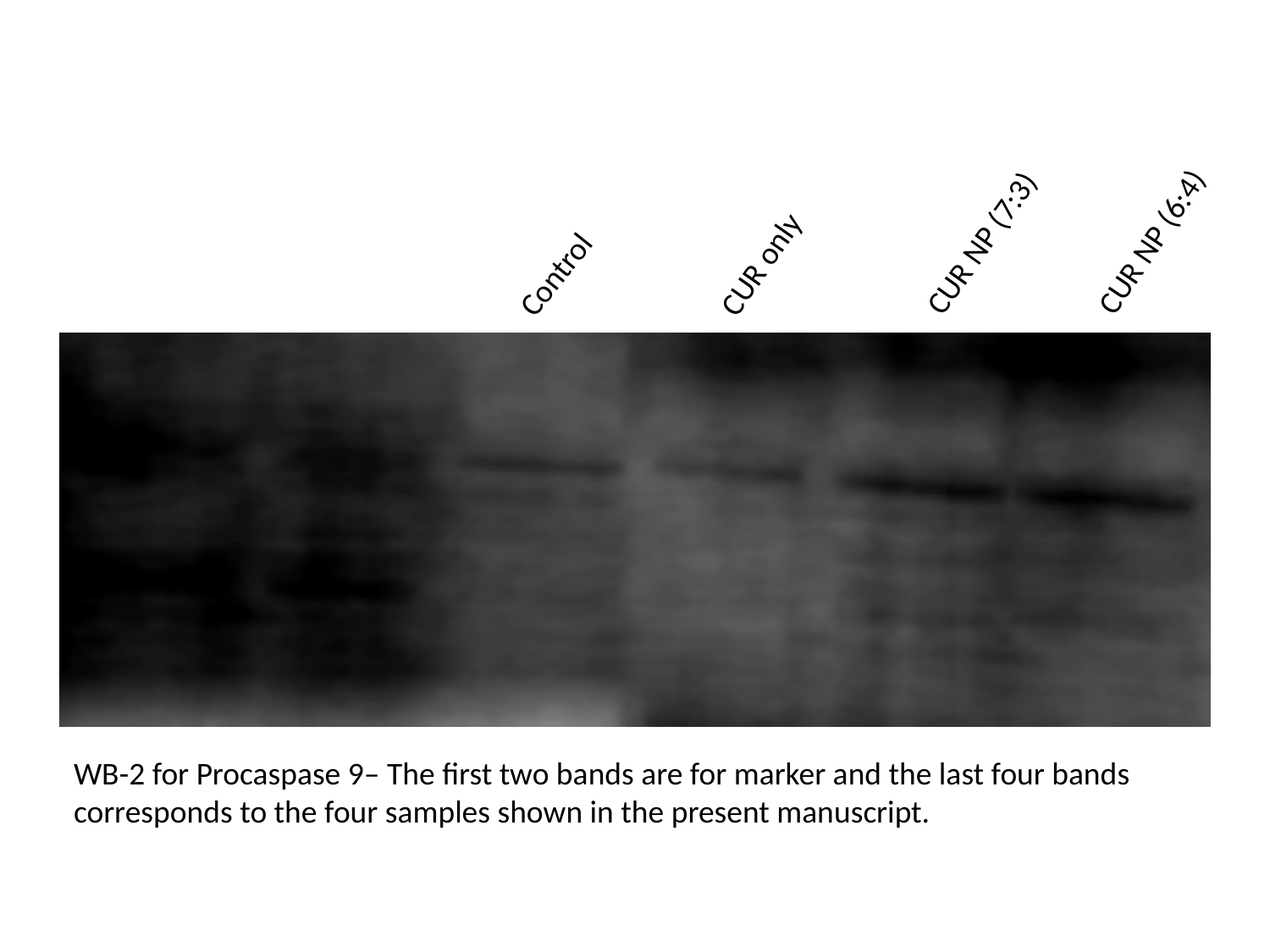

CUR NP (6:4)
CUR NP (7:3)
CUR only
Control
WB-2 for Procaspase 9– The first two bands are for marker and the last four bands corresponds to the four samples shown in the present manuscript.
